# Perceived need for care and stigma experiences among individuals with methamphetamine-related admissions to inpatient mental health wards

**DOI:** 10.1186/s12954-023-00827-5

**Published:** 2023-08-02

**Authors:** Lochlan Wilson, Niketa Karnik, Jacquelyne Y. Wong, Lyra Barchet, Jitender Sareen, Ahmed Jwely, Nathan Nickel, Geoffrey Konrad, Joshua Nepon, James M. Bolton

**Affiliations:** 1 Department of Psychiatry, College of Medicine, Faculty of Health Sciences, University of Manitoba, Winnipeg, MB Canada; 2 Department of Community Health Sciences, College of Medicine, Faculty of Health Sciences, University of Manitoba, Winnipeg, MB Canada

**Keywords:** Methamphetamine, Canada, Perceived need, Stigma

## Abstract

**Background:**

There are gaps in our understanding of treatment needs among people who use methamphetamine. We examined the demographics, perceived treatment needs, barriers to accessing care, and stigma experienced by an inpatient sample of people who use methamphetamine.

**Methods:**

This study surveyed a convenience sample of patients admitted to psychiatry wards with a history of methamphetamine use in Winnipeg, Canada, between May 1 and July 31, 2019. The Perceived Need for Care Questionnaire (PNCQ-9) was used to assess treatment needs and barriers to care, and the Substance Use Stigma Mechanisms Scale (SU-SMS) was used to assess enacted, anticipated, and internalized stigma. Prevalence rates of perceived need, stigma, and demographic variables were determined.

**Results:**

A total of 103 potential participants were identified, with 34 completing the survey. The most common age group was 21–30 years of age (41.2%); an approximate equal number of men and women; and almost all were single and never married (91.1%). Rates of perceived need for care were very high across all treatment types, including 91% identifying a need for medication treatment for their mental health or substance use. Despite the majority receiving care across the seven types of care described in the PNCQ-9, most felt they did not receive enough care. Unmet need for care was therefore high in many categories, including rates of 87% for counselling and skills training. The most common barriers to having needs met were a desire to self-manage substance use, and not receiving care after asking for help. Almost all participants reported experiencing stigma (94%). Stigma from family was endorsed significantly more than stigma from health care providers (*p* = 0.005).

**Conclusions:**

The average hospitalized person who uses methamphetamine in this sample is young, single, and has not completed any post-secondary education. High rates of perceived treatment need suggest an awareness of problems with methamphetamine, yet most interventions are perceived as inadequate. People who used methamphetamine felt highly stigmatized, particularly by their family members.

*Trial registration* Registered with the Health Research Ethics Board at the University of Manitoba (Number HS22605 (H2019:072), renewed February 14, 2022).

## Background

Harms related to methamphetamine use have rapidly become a major public health concern. While the overall prevalence of methamphetamine use in Canada remains low at an estimated 1-year prevalence of 0.2%, certain regions have noted a rapid increase in its availability [1]. In Manitoba, presentations to Winnipeg emergency departments (ED) for methamphetamine-related concerns increased by 1700% between 2013 and 2019 [2]. The concern regarding the exponential rise in prevalence is compounded by the severe consequences observed among people who use methamphetamine. A recent meta-analysis revealed that 36.5% of people who use methamphetamine develop symptoms of psychosis, a figure far higher than with any other illicit drug [3]. Local data show that Manitobans who used methamphetamine were 43.7 times more likely to be diagnosed with a psychotic disorder within 12 months of their first methamphetamine-related contact with the healthcare system than the general population [4]. It can be difficult to differentiate acute symptoms of psychosis related to methamphetamine use from those related to a chronic primary psychotic disorder [5]. It may appear that methamphetamine can induce symptoms in otherwise healthy individuals, but a more nuanced view may be that it reveals a vulnerability to psychosis that was already present, while longer-term methamphetamine use may also increase vulnerability to developing symptoms of psychosis [5]. While some of these presentations featuring psychosis become chronic, even those associated solely with methamphetamine intoxication frequently last several days following last use, greatly complicating disposition planning in the ED. Individuals who are intoxicated with methamphetamine or who are experiencing symptoms of psychosis following methamphetamine use show behaviours that may be difficult to predict [6], though the view that methamphetamine use leads to violent behaviour has not been causally borne out in literature [7]. However, people who use methamphetamine may be more likely to exhibit challenging behaviours that can escalate to violence based on the response of those around them, and thus may be equated with violence [7]. People who use methamphetamine are more likely to be victims of violence, especially if they are unhoused [7]. Methamphetamine use disorders have a high mortality rate [8] and, in Manitoba, methamphetamine has been found to be present in an increasing number of cases of opioid-related death, rising from 4 to 25% between 2014 and 2017 [9]. Across Canada, stimulants were involved in over half of deaths by accidental apparent opioid toxicity between 2018 and 2022 (56–64%) [10]. There is a high rate of relapse among people who use methamphetamine after substance use disorders treatment, with one study showing that 61% of people discharged from a substance use treatment system used methamphetamine again within 1 year [11]. There is growing evidence within stimulant use disorder research to suggest that agonist therapy with prescription psychostimulants can promote abstinence from recreational stimulants [12]. However, the majority of these studies exclude participants with psychiatric comorbidities like mood or psychotic disorders. Furthermore, even when people who are motivated to seek medications to manage their stimulant use disorder, there may be a limited number of providers willing to prescribe in their area. People who use methamphetamine may also have their own outcomes of interest when evaluating potential treatments, highlighting the importance of qualitative research in this area [13]. There is a lack of strong evidence on pharmacological management of acute psychiatric concerns in the context of methamphetamine intoxication.

While there is growing concern about the prevalence of methamphetamine use, there is a limited understanding about whether people who use methamphetamine feel their use is problematic and if they perceive a need for treatment. A study showed that 84.9% of women in Appalachia who use methamphetamine reported feeling like they had a problem with drugs, and 62.9% felt they needed treatment immediately [14]. Perceived need for treatment has been extensively studied in other mental disorders and has been found to be an important factor predicting service use [15–17]. While it is estimated that 20% of Canadians meet criteria for a mental disorder, a 2005 study by Sareen et al. reported that only 8.3% of Canadians seek treatment for mental health or emotional problems [15]. Another study showed that 65% of Canadians with substance use disorders did not seek services or supports for their mental health, and when they did, they were less likely to seek formal supports or specialist care [18]. This gap is often explained by barriers to care, including stigma. A study that examined perceived need for care among street-involved people who used illicit drugs showed that the majority of individuals reported one or more unmet needs in the past year, and that housing instability was significantly related to more than one unmet need [19]. A study conducted in Australia found that people with substance use disorders were less likely to report a need for care than people with affective or anxiety disorders [20]. Knowledge about perceived need can also be used to improve the patient-centeredness of care. Compared to other substances, barriers to accessing treatment for methamphetamine use have a relatively small body of research. One meta-analysis examined barriers to accessing care reported by people who use methamphetamine found only 11 relevant studies on barriers to methamphetamine treatment [21]. It showed that psychosocial barriers to treatment were the most commonly reported, such as feeling that treatment was unnecessary, embarrassment or stigma, wanting to withdraw on their own, and being concerned about privacy [21]. Despite this study, there remains very little research examining the perceived need, and barriers to care experienced by people who use methamphetamine.

There is also a gap in our knowledge about how stigma affects people who use methamphetamine and the care they seek. Studies have conceptualized different forms of stigma, such as enacted stigma, anticipated stigma, and internalized stigma based on their source and the experience of the person stigmatized [22]. Smith et al. provided the following definitions of stigma as it relates to substance use; *Enacted Stigma* includes ‘personal experiences of stereotyping, prejudice, and/or discrimination from others’, while *Anticipated Stigma* includes expectations of these experiences in the future [22]. *Internalized Stigma* refers to ‘the endorsement and application of negative feelings and beliefs’ about people who use substances to themselves [22]. A systematic review found that the attitudes of healthcare workers towards people with substance use disorders is generally negative [23]. Qualitative interviews have been used to examine perceptions of stigma in people who inject drugs in California, where it was found that stigma impacted healthcare service utilization [24]. A study of people who inject drugs found that many experienced being treated as ‘drug-seeking’ when requesting management of pain and other symptoms, which could lead to inadequate pain control and unmitigated withdrawal symptoms [25]. Others reported being treated with suspicion, which can contribute to negative experiences of the hospital environment [25]. Another study noted that self-reliance, minimization of the severity of substance use problems, fear of social consequences, and financial and structural barriers were common reasons why people in the USA did not get substance use disorder treatment [26]. A recent study found that having a family member or friend who used methamphetamine did not reduce stigmatizing beliefs about methamphetamine use, which contrasted with previous research about other substances [27]. There have been studies aimed at reducing stigma from family members in other settings and with other stigmatized conditions like HIV [28, 29] and schizophrenia [30]. While there have been more studies on interventions to reduce stigma around substance use from healthcare providers and systems [31], less is known about ways stigma experienced from the family members of people who use substances can be mitigated.

In order to address these gaps in knowledge and further an understanding of methamphetamine use, this study sought to determine the rates of perceived unmet needs for care in this group, the barriers that people who use methamphetamine face in accessing care, and the prevalence of enacted, anticipated, and internalized stigma in this group. A secondary aim was to identify the demographics of people who use methamphetamine. We expected the prevalence of enacted, anticipated, and internalized stigma among people who use methamphetamine to be high, based on stigma reported by populations who use other substances. We also anticipated that the number of perceived needs will be high in this population, and that they will identify several barriers in accessing health care.

## Methods

### Study setting and population

This study was a convenience sample of patients admitted to adult, non-forensic psychiatry wards at the PsycHealth Centre in Winnipeg, Canada, whose presentations featured methamphetamine use between 1 May to 31 July 2019. The PsycHealth Centre is located at the largest tertiary hospital in Manitoba and contains approximately 100 adult inpatient beds. It was chosen for its central location in the province and large number of acute adult inpatient beds. Other hospitals in Winnipeg were not included due to personnel limitations. There is an addictions medicine consultation service available at this hospital, with services focused on guidance on acute withdrawal management, as well as maintenance or initiation of pharmacotherapy for substance use, primarily for alcohol and opioid use disorders. There is also a rapid access to addictions medicine (RAAM) clinic in a nearby building associated with the hospital that can be accessed by the general public, as well as inpatients that offers advice, counselling, medications, and harm reduction supplies [32]. However, provision of harm reduction information and supplies was not a routine part of inpatient care or discharge planning at the time of data collection. At the time of data collection and writing, there were no publicly funded supervised consumption sites in the province of Manitoba. Inclusion criteria for participants were having been admitted to a non-forensic ward for presentations that included methamphetamine use (as per the patient or per positive urine drug screen) and being 18 years of age or older. Patients were screened for eligibility by psychiatric nurses involved in their care, or by their treating psychiatrist. A diagnosis of methamphetamine use disorder or methamphetamine-induced psychosis was not required. If a patient denied methamphetamine use but was still agreeable to complete the survey, their results were included if there was collateral information or a positive urine drug screen, confirming that they had recently used methamphetamine. Urine drug screens were not routinely collected on all participants as this was not in keeping with routine clinical practice. Potential participants were excluded if they did not have capacity to consent to participate of if they were deemed inappropriate for the study by the care team; this comprised individuals where the safety of the interviewer would be a concern, as well as patients whose clinical course might have been negatively impacted by sensitive questions, as determined by the treating psychiatrist involved in their care. Informed consent was gained by staff before the questionnaires were administered. The study was approved by the Health Research Ethics Board of the University of Manitoba (Registration Number HS22605 (H2019:072)).

### Study design and data collection

This study employed a cross-sectional design with each element of the survey administered in one sitting with data collected between May 1 and July 31, 2019. Patients were approached by a member of the care team, usually a nurse, and asked if they would be agreeable to a survey conducted by a research team member. If the patient was interested in completing the survey, informed consent was gained verbally. No financial, material, or social incentives for participating were offered. Surveys were administered by one of two members of the research team, occasionally with a member of the care team if safety was a concern. The two team members who administered surveys were a medical student and a psychiatric resident physician at the time of data collection.

### Measures

Patients first filled out demographic information, including Age (18–20, 21–30, 31–40, 41–50, 50+), Gender (man, woman, non-binary), Education level (no high school, some high school, high school, some post-secondary, post-secondary), Marital status (single, divorced, married/common law), length of methamphetamine use (< 6 months, 6 months to 1 year, 1 to 5 years, more than 5 years), and length of time since previous substance use treatment (which was broadly defined to include any inpatient, outpatient or residential treatment for substance use, as well as any pharmacotherapy related to reducing use or harms associated with substance use), if any.

Perceived need, treatment seeking, and barriers to accessing care were assessed using a modified form of the Perceived Need for Care Questionnaire (PNCQ-9) [33]. The PNCQ-9 uses a four-stage design that enquires about the services received by subjects, the types of interventions they received, if they received enough care, and barriers to care within the last 12 months [33]. The PNCQ covers 7 types of care: Information, Medication, Hospital Care, Counselling, Skills Training, Social Interventions, and Harm Reduction [33]. A description of these types of care can be found in Table 1. Some questions were re-formatted to suit the structure of the local healthcare system.

**Table 1:** Descriptions of the 7 types of care included in the Perceived Need for Care Questionnaire (PNCQ-9)

Type of care	Description
Information	Information about treatments or available services
Medication	Prescribed medication for emotions, mental health, or substance use
Hospital care	Inpatient admission overnight or longer, not including emergency room visits
Counselling	Any kind of help to talk through problems
Social interventions	Help sorting out practical issues such as income support, emergency shelters, subsidized housing
Skills training	Help to improve your ability to work, to care for yourself, to use your time or to meet people
Harm reduction	Services designed to reduce the risk of harm related to using drugs, such as needle exchange

Stigma was measured using the Substance Use Stigma Mechanisms Scale (SU-SMS) [22]. The SU-SMS is a questionnaire based on stigma theory that has been tested for validity and reliability [22]. It includes three sections, with enacted stigma, anticipated stigma, and internalized stigma related to substance use being measured. Participants respond to questions about stigma on a 5-point Likert-type scale: (1) never, (2) not often, (3) somewhat often, (4) often, and (5) very often.

### Statistical analysis

Completed surveys were entered into a database and analysed using SPSS. Descriptive statistics were determined for the demographic information, stigma scores (for enacted, anticipated, and internalized stigma), and the number of perceived unmet needs reported by each participant. Stigma source subscales (family vs. healthcare) were calculated by taking the averages of the enacted and anticipated stigma responses to questions that referred to healthcare workers and to the questions referring to family members. Unmet need was calculated as the difference between the perceived need for a given type of care, and the endorsement of receiving enough care.

## Results

**Fig. 1:**
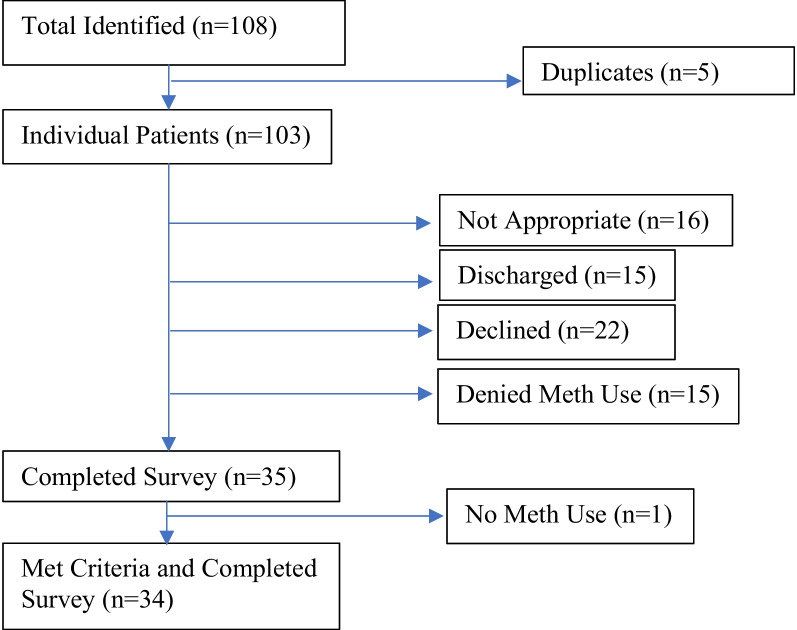
Depiction of the study population. Participants who were flagged as eligible for the survey multiple times erroneously were removed. Patients were deemed not appropriate for the survey if their treating psychiatrist determined that they would not be able to tolerate the survey, if there were safety concerns for the study personnel, or if their clinical course would be negatively affected by sensitive questions. Some potential participants were discharged before the survey could be administered. Some potential participants endorsed methamphetamine use but declined to be surveyed, while others denied having used methamphetamine and declined to be surveyed. One participant who had not used methamphetamine completed the survey and was removed from the final sample

A total of 108 patients were identified by the care team as having recently used methamphetamine. Three of these patients were repeat admissions who had either completed the survey or refused to participate in an earlier methamphetamine-related admission, and 2 patients were erroneously flagged a second time during the same admission each, giving a total of 103 unique potential participants. Twenty-two potential participants declined the survey. Fifteen were discharged before they were able to complete the survey, and another 16 were deemed not appropriate for the study by members of the care team for issues such as irritability or aggression. Finally, 15 of the identified patients denied methamphetamine use. This left a group of 35 participants who completed the survey. One patient was removed from calculation as the participant had been incorrectly assessed as having used methamphetamine (confirmed by a negative urine drug screen). A visual representation of the sample can be found in Fig. 1.

### Demographics

The largest age group was the 21–30 group, which contained 41.2% of participants, followed by the 31–40 group, which contained 35.3%. There were an almost equal number of men and women who took part in the study (15 men, 18 women), and one participant identified as gender non-binary. With regard to education, 47.1% of participants had not completed high school, while 29.4% had completed high school and 23.5% had at least some post-secondary education. Almost all participants were single and had never been married (91.1%). Many had received previous treatment for substance use (67.6%). The length since last substance use treatment ranged between 1 month ago and 18 years ago. 44.1% of participants had been using methamphetamine for less than 1 year, including 11.7% for whom it was their first time using. A full table of demographic results can be found in Table 2.

**Table 2:** Demographic and methamphetamine use information for participants (*n* = 34)

Characteristic	*n* (%)
*Age*	
18–20	3 (8.8)
21–30	14 (41.2)
31–40	12 (35.3)
41–50	3 (8.8)
> 50	2 (5.9)
*Gender*	
Man	15 (44.1)
Woman	18 (52.9)
Non-binary	1 (2.9)
*Education level*	
No high school	6 (17.6)
Some high school	10 (29.4)
High school	10 (29.4)
Some post-secondary	7 (20.6)
Post-secondary degree	1 (2.9)
*Marital status*	
Single	31 (91.2)
Married/common law	1 (2.9)
Separated/divorced	2 (5.9)
*Length of methamphetamine use*	
First use	4 (11.8)
Less than 6 Months	6 (17.6)
6 Months to 1 year	5 (14.7)
1–5 Years	12 (35.3)
More than 5 years	7 (20.6)
*Previous treatment*	
Yes	23 (67.6)
No	11 (32.4)

### Perceived need

Rates of perceived need for methamphetamine treatment were very high across all categories (Table 3). Ninety-one per cent identified a need for information about methamphetamine use, medication treatment, and counselling. Skills training, defined as help to improve one’s ability to work, to care for oneself, or to use one’s time or to meet people, was identified as a need by 67.6% of respondents (*n* = 23), but 87% of these (*n* = 20) felt they did not receive enough help. 44.1% (*n* = 15) of participants cited a perceived need for harm reduction services. The majority of participants reported receiving treatment in the areas of information, medication, hospital management, and social interventions. However, aside from medication treatment and harm reduction, the majority of respondents reported that they did not receive enough care in all treatment categories. This disparity between a perceived need and receiving enough care for that need resulted in high rates of unmet need for certain types of care. Of the 31 participants who cited a need for counselling, 27 (87.1%) reported that they did not receive enough. Similarly, 20 (87%) of the 23 who identified a need for skills training felt they did not receive adequate training. Nine participants (29%) had 2 or fewer unmet needs, while 13 (42%) had 3–4 unmet needs, and the remaining 9 participants (29%) had 5 or more unmet needs.

**Table 3:** Rates of perceived need and unmet need for the seven types of care from the PNCQ-9

	Information *n* (%)	Medication *n* (%)	Hospital care *n* (%)	Counselling *n* (%)	Social interventions *n* (%)	Skills training *n* (%)	Harm reduction *n* (%)
Perceived a need for care	31 (91.2)	31 (91.2)	30 (88.2)	31 (91.2)	27 (79.4)	23 (67.6)	15 (44.1)
Received care	22 (64.7)	28 (82.4)	30 (88.2)	12 (35.3)	20 (58.8)	5 (14.7)	12 (35.3)
Felt they received enough care	15 (48.4)	21 (67.7)	11 (36.7)	4 (12.9)	9 (33.3)	3 (13.0)	10 (67.7)
Unmet need for care^a^	16 (51.6)	10 (32.3)	19 (63.3)	27 (87.1)	18 (66.7)	20 (87.0)	5 (33.3)

The types of care are defined in Table 1 (*n* = 34)

Denominator for ‘perceived a need for care’ and ‘received care’ was the full sample (*n* = 34). Denominator for ‘felt they received enough care’ and ‘unmet need for care’ was the ‘*n*’ for ‘perceived a need for care’ within that treatment category

^a^Unmet Need for Care was calculated by subtracting the number of people who felt they received enough care from the number that perceived a need for that type of care

Barriers that were not included in the questionnaire (the ‘Other’ response) were the most commonly cited causes for not receiving enough care by study participants. Examples of barriers to receiving care for each type of care are provided in Table 4.

**Table 4:** Examples of free text explanations of ‘Other’ barriers to receiving adequate care from the seven areas of care described in the PNCQ-9

Type of care	Example of response
Information	“Had the basics, needed higher level information” “I think I got less information because I got angry at the program staff”
Medication	“Not enough resources in Northern [Manitoba] community. I always have to fly to see my doctor” “Hard to get good follow-up after discharge, so I couldn't get the meds”
Hospital Care	“Not enough access to culturally appropriate care (no sweat lodge at [the hospital])” “Avoided hospital care because of trust issues and paranoia”
Counselling	“not a good fit with therapist, lack of trust in psychiatrist and counsellor” Scared I would get in trouble if I opened up more”
Social Interventions	“Working round-the-clock made it impossible to properly access resources” “Aged out of [Child and Family Services Care], turned away due to meth use, no specific med-focused resources”
Skills Training	“Caring for sick grandma and kids so I don't have time to do skills training” “Felt discriminated against when it came to opportunities. Also emotions got in the way”
Harm Reduction	“Doctor didn't give me leave to go get harm reduction supplies” “Not enough resources to go around in the city”

The second most cited barrier to receiving care was a preference to manage the various aspects of one’s substance use by oneself, followed by having asked for help but not receiving it.

### Stigma

Overall, 32 out of 34 participants reported experiencing at least one form of stigma due to their use of substances. Within each category of stigma, 32 reported experiencing enacted stigma, 32 reported experiencing anticipated stigma, and 32 reported internalized stigma. Stigma experienced from family members was higher than that from healthcare workers on a stigma source subscale (3.4 vs. 2.8 on a scale of 1–5, *p* = 0.005). The difference between anticipated stigma from family members and healthcare workers (average of 2.9 and 2.9 on a scale of 1–5, respectively) was not statistically significant (*p* = 0.07).

## Discussion

This study presents novel information about perceived need and stigma among people who use methamphetamine admitted to psychiatric wards. The patient population who uses methamphetamine is difficult to capture in a survey study, so even with a small sample size our results were able to paint a clearer picture of who is using methamphetamine. Out of our sample, the average hospitalized person who uses methamphetamine in this sample is between 21 and 30, single, has not completed any post-secondary education, and has received treatment for substance use in the past. Many of those hospitalized have also only recently started using methamphetamine, with 44% having started their methamphetamine use in the past year. This is useful information when it comes to targeting interventions towards people who use methamphetamine.

From our observations, most people who use methamphetamine have experienced enacted stigma, anticipate experiencing future stigma, and have internalized stigma about their methamphetamine use. The external sources of stigma come from both family and healthcare workers. Distinguishing the sources of stigma for people who use methamphetamine could direct research into ways to reduce stigma against people who use this substance. There is some research into the effectiveness of various methods to reduce the stigma surrounding substance use within the healthcare system including education regarding less stigmatizing terminology [34], incorporating reflection tasks into medical training, and tailoring medical students’ clinical experiences to involve populations who use substances [31], but there is a gap in our knowledge about how to reduce stigma coming from the families of people who use substances.

Other important considerations when targeting interventions to people who use methamphetamine are the type of interventions that they perceive they need. Counselling and skills training were commonly cited as being unmet needs. Medications were perceived as a need by 91.2% (*n* = 31) of participants, and while there is growing evidence to support agonist therapy in people who use stimulants, these strategies have not been widely put into practice in a standardized fashion [12]. Harm reduction has been shown to be an effective intervention in illicit substance use [35], but relatively fewer participants identified it as a need compared to other types of care. This could highlight a gap between the evidence and perceptions of the importance of harm reduction. Some participants may not have been familiar with the range of harm reduction services or may have not felt comfortable asking for these services due to past discrimination. Participants in another Canadian study reported feeling like asking for harm reduction supplies like clean syringes would attract suspicion or even risk involuntary discharge for violating hospital policies [25]. While some hospitals in North America have implemented harm reduction services such as distributing harm reduction kits [36] or opening a safe consumption site open to inpatients [37], there were few interventions available at the hospital where this study took place during the time of sampling.

Overall, most patients perceived multiple unmet needs. Mojtabai and Crum found in a longitudinal study that perceived need was associated with future engagement in substance use services among patients with substance use disorder [26], so it could be that a patient who has perceived a need has considered changing their behaviour in ways such as seeking treatment. Since many of these participants interviewed in hospital perceived a need for care, perhaps this location is an important area for the future allocation of resources for efforts to encourage behavioural change among people who use methamphetamine. No formal analysis on the barriers to care section of the PNCQ was conducted due to the low sample size, but a future study concerning barriers to people who use methamphetamine accessing healthcare services specifically using a qualitative approach would be useful to guide public policy changes and future interventions.

### Strengths and limitations

A strength of this study was acknowledgement of the compounding forms of enacted, anticipated, and internalized stigma. However, this did not use measures to account for other intersecting forms of stigma that can influence treatment in hospital settings, such as sexism, racism, homophobia, transphobia, ageism, or classism. These intersecting forms of discrimination could influence participants perceived needs and barriers in a way that this study could not account for. The Chief Public Health Officer’s Report on the State of Public Health in Canada from 2019 focused on stigma as a public health issue, and is a useful resource to the academic community as it describes the experiences of various stigmatized groups, the effects of stigma on health, as well as ways to make health systems more inclusive [38].

There were limitations that impact the results of this study. First, the sample size was small due to a short study timeline and low engagement from patients. We found that patients recovering from methamphetamine withdrawal were often tired, irritable, and occasionally paranoid, limiting capacity to consent and participate in the survey. Roughly 108 potential participants were flagged by the care team as having used methamphetamine, but only 34 agreed to and successfully completed the survey. This reduced our ability to conduct statistical analysis and limits generalizability to groups that were excluded. Second, the design of the study could have resulted in a sample that underrepresented certain subgroups of patients who use methamphetamine including patients with significant intellectual disabilities, low frustration tolerance, or patients experiencing ongoing symptoms of psychosis like disorganization. There may have been common characteristics among those who declined to participate that could have made the sample less representative of the population, potentially limiting generalizability of the findings. For example, a potential participant who could not tolerate the length of the interview might have reported different perceived needs and stigma compared to the subset of the subset who did complete the survey. The staff who administered the survey may have been perceived as being healthcare workers, potentially confounding the participants’ responses to questions regarding healthcare workers as a source of stigma. Possible ways to improve engagement among this group could include using shorter measures, increasing length of data collection, and incentivization of participation where it is ethical. Another limitation is that this study was limited to an inpatient population and thus cannot directly address perceived need and stigma among people who use methamphetamine in the community.

Psychosis is a symptom experienced by many people who use methamphetamine [3, 4] and a common indication for inpatient admission. This study attenuates the potential of confounding by indication by surveying a population admitted to hospital, who would have been admitted for psychiatric concerns such as mood or psychotic disorders that may be related to methamphetamine use. If these admissions were directly related to help-seeking for methamphetamine-related problems, rates of perceived need could be artificially inflated and biased by this subgroup. However, most admissions were because of psychosis or mood disorders, and therefore, once participants were able to complete a survey, it potentially provided a more unbiased examination of perceived need for methamphetamine treatment.

### Future directions

Future studies examining methamphetamine use in an inpatient population will have several issues to contend with when trying to reach this population. First, there can be a very short timeline between a patient admitted to hospital and being well enough to complete the survey prior to being discharged. There are also issues with subsets of the population that cannot complete the survey or may be too agitated to be safely interviewed by research staff. The effects of methamphetamine intoxication, such as behavioural disinhibition and anxiety, and withdrawal, such as somnolence [39], makes accurate survey completion more challenging. Lastly, many patients either denied using methamphetamine, or were incorrectly labelled as having used methamphetamine. This is a difficult area to parse, as there is a high level of stigma people who use methamphetamine which could increase denial of use, but there is also a lot of polysubstance use that makes the use of methamphetamine harder to confirm. These causes of confusion can make it harder to accurately say whether methamphetamine is the cause of an admission.

## Conclusion

This study shows that the prevalence of both stigma and perceived needs is high among people admitted to hospital for methamphetamine-related problems. This study also identifies some key obstacles in reaching this population with which future studies would have to contend. The field of study of stigma and methamphetamine use is ripe for future studies, such as examining the differences in the sources of stigma and ways to reduce their impact. Further research on the barriers that people who use methamphetamine face when trying to access care would have implications for disposition planning and programme development. A study investigating the effect that stigma has on perceived need and treatment seeking could shed light on a potential connection that could be targeted in future interventions.

## Data Availability

The datasets used and/or analysed during the current study are available from the corresponding author on reasonable request.

## Abbreviations

ED: Emergency Department
PNCQ-9: Perceived Need for Care Questionnaire
SU-SMS: Substance Use Stigma Mechanisms Scale
